# The Relationship between Sclerostin and Kidney Transplantation Mineral Bone Disorders: A Molecule of Controversies

**DOI:** 10.1007/s00223-024-01261-w

**Published:** 2024-07-30

**Authors:** Baris Afsar, Rengin Elsurer Afsar, Yasar Caliskan, Krista L. Lentine

**Affiliations:** 1 Department of Nephrology, School of Medicine, Suleyman Demirel University, Isparta, Turkey; 2 Department of Nephrology, Saint Loui University, Saint Louis University Hospital, Saint Louis, MO USA

**Keywords:** Bone, Chronic kidney disease, Kidney transplantation, Sclerostin, Vascular calcification

## Abstract

Kidney transplantation is the most effective treatment option for most patients with end-stage kidney disease due to reduced mortality, decreased cardiovascular events and increased quality of life compared to patients treated with dialysis. However, kidney transplantation is not devoid of both acute and chronic complications including mineral bone disorders (MBD) which are already present in patients with chronic kidney disease (CKD) before kidney transplantation. The natural history of MBD after kidney transplantation is variable and new markers are needed to define MBD after kidney transplantation. One of these promising molecules is sclerostin. The main action of sclerostin is to inhibit bone formation and mineralization by blocking osteoblast differentiation and function. In kidney transplant recipients (KTRs), various studies have shown that sclerostin is associated with graft function, bone parameters, vascular calcification, and arterial stiffness although non-uniformly. Furthermore, data for inhibition of sclerostin with monoclonal antibody romosozumab for treatment of osteoporosis is available for general population but not in KTRs which osteoporosis is highly prevalent. In this narrative review, we have summarized the studies investigating the change of sclerostin before and after kidney transplantation, the relationship between sclerostin and laboratory parameters, bone metabolism and vascular calcification in the context of kidney transplantation. We also pointed out the uncertainties, explained the causes of divergent findings and suggest further potential study topics regarding sclerostin in kidney transplantation.

## Introduction

Kidney transplantation is the treatment of choice for most patients with end-stage kidney disease due to reduced mortality, decreased cardiovascular events, and increased quality of life compared to patients treated with dialysis [1]. However, kidney transplantation is not devoid of both acute and chronic complications including acute rejection, infections, cardiovascular events, and chronic allograft failure. With the development of new immunosuppressive regimens and improved biomarkers for rejection and graft injury surveillance, acute rejection episodes decreased but long-term kidney allograft survival has not improved in parallel and patient mortality exceeds that of the general population [2]. Non-immune complications of kidney transplantation include infections, malignancy, cardiovascular disease, and mineral bone disorders (MBD). To improve long-term patient survival, new management strategies are needed. One of these strategies is to find new biomarkers to facilitate early diagnosis and management of long-term, non-immune complications.

Mineral and bone disorders which are already present in both dialysis dependent and independent chronic kidney disease (CKD) patients may persist after kidney transplantation [3]. Although the name CKD–MBD suggest the problem is on bone metabolism, CKD–MBD is a systemic disorder including: (i) abnormalities of calcium, phosphorus, parathyroid hormone (PTH), or vitamin D metabolism; (ii) abnormalities in bone turnover, mineralization (e.g., osteoporosis), bone volume and (iii) vascular or other soft tissue calcification [4] or variable combinations of these. Indeed, in KTRs, crosstalk between kidney, arteries and bone via molecules and signaling pathways play a role in the development of CKD–MBD [5, 6] and sclerostin is one of these of molecules. After kidney transplantation, various pathologies such as hypercalcemia, hypophosphatemia, persistent hyperparathyroidism, de novo hyperparathyroidism, osteomalacia, osteopenia, osteoporosis, and vascular calcification may still be observed [6, 7]. Indeed, sclerostin has been considered as a new biomarker in CKD–MBD [8] which has been associated with various clinical, laboratory, and bone parameters, but with conflicting results. There are also various studies investigating the role of sclerostin in various aspects (parameters of bone metabolism, bone volume, bone remodeling, vascular calcification etc.) in kidney transplant recipients (KTRs) with divergent results. To advance understanding of the role of sclerostin in MBD physiology in the context of kidney transplantation, we conducted a review of studies investigating the changes in sclerostin before and after kidney transplantation, the relationship between sclerostin and laboratory parameters, as well as bone metabolism and vascular calcification in the context of kidney transplantation.

## Methods

**Fig. 1:**
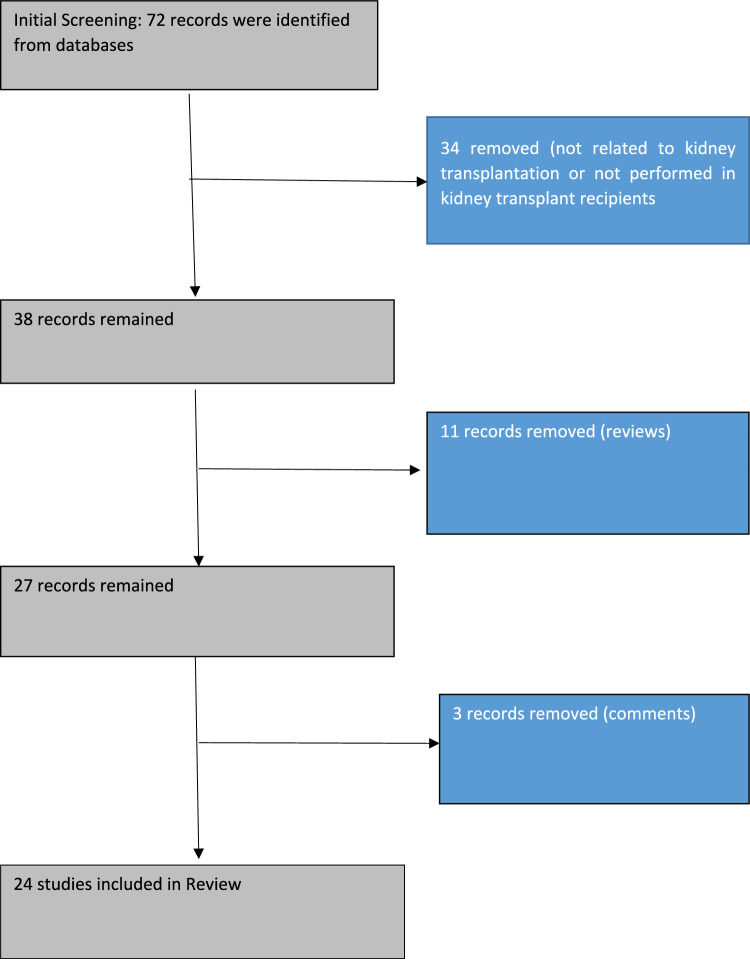
Flow chart of included studies

We performed (BA and REA) review of the literature in English language until March 2024 using major online databases (PubMed, Scopus, Web of Science, Cochrane Library and Google Scholar) using the key words “sclerostin renal transplantation,” “sclerostin and kidney transplantation,” “sclerostin and kidney graft failure,” “sclerostin cardiovascular disorders and renal transplantation,” “sclerostin cardiovascular disorders and kidney transplantation,” “sclerostin bone mineral disorders and kidney transplantation,” “sclerostin bone mineral disorders and renal transplantation.” After screening of the studies, only studies in the context of kidney transplantation were included. We included both cross-sectional, longitudinal, and interventional studies. The flow chart of included studies are shown in Fig. 1.

## Results

The studies regarding sclerostin measurements, correlates of clinical, biochemical, bone parameters, and vascular calcifications with sclerostin in the context of kidney transplantation are summarized in Table 1. The studies are heterogeneous with respect to design, patient’s characteristics, follow-up period, and outcome measures. Some studies are cross-sectional [9–11] and some studies are longitudinal [12–16]. Furthermore, sclerostin measurements were measured before in kidney transplant in some studies (baseline sclerostin measurements) [17, 18] others measured sclerostin levels at post-kidney transplantation [19–21] and others measured sclerostin levels both baseline (before kidney transplantation) and after kidney transplantation [22, 23].

**Table 1:** Studies summarizing sclerostin assessment in the context of kidney transplantation

References	Methods	Specific results for sclerostin levels and bone parameters	Graft function	Sclerostin hemodynamic alterations MACE, mortality
Bonani et al. [23]	– Serum sclerostin measured before and during the first year of kidney transplantation in 42 KTRs and its association with parameters of bone mineral metabolism and with bone mineral density – Participants receive vitamin D (800 IU/day) and half of the patients were treated with denosumab (60 mg subcutaneous every 6 months) as part of the study protocol – Sclerostin was measured by ELISA (TecoMedical)	– Pre-transplant serum sclerostin elevated in all patients (61.8 ± 32.3 pmol/l) but within 15 days after kidney transplantation, sclerostin levels dropped to 21.0 ± 14.7 pmol/l and subsequently increased to 23.8 ± 14.9 and 28.0 ± 16.8 pmol/l after 6 and 12 months, respectively (*P* < 0.001) – No correlation was found between post-transplant sclerostin and bone mineral density – Tacrolimus, cyclosporine, calcium, bisphosphonates, cinacalcet, denosumab did not significantly associate with the post-transplant sclerostin	No association between GFR and sclerostin trajectory	NDA
Tomei et al. [112]	– In 19 post-menopausal KTRs and in 12 age matched post-menopausal CKD patients serum levels of sclerostin, DKK1, N-terminal procollagen type 1, bone-specific alkaline phosphatase, and serum C-terminal telopeptides of type I collagen measured – Sclerostin levels were measured with an enzyme immunoassay (Biomedica)	– Sclerostin similar among KTRs and CKD (absolute values are not given) – Sclerostin positively correlated with serum phosphorus in multivariate analysis	– Sclerostin negatively correlated with estimated GFR in multivariate analysis	NDA
Evenepoel et al. [16]	– Coronary artery calcification (CAC) and aortic calcifications measured by CT in 268 prevalent KTRs at baseline and re-measured in 189 patients after a median follow-up of 4.4 years – Baseline serum sclerostin levels were assessed on stored blood samples – Serum sclerostin was determined with an ELISA (TecoMedical)	– The median sclerostin level was 0.84 (0.62–1.10) ng/ml	– GFR tended to be lower in KTRs with sclerostin above median vs. below median (48.7 vs. 54.2 ml/min/1.72 m^2^, respectively, *P* 0.06)	– There is positive correlation between CAC, CAC progression (occurred in 47.1% KTRs), and baseline sclerostin in univariate analysis which was lost in multivariate analysis – Baseline aortic calcification was positively correlated with sclerostin in univariate analysis which was reversed in multivariate analysis (lower sclerostin higher aortic calcification) – Progression of aortic calcification (occurred in 45.5% KTRs) was not associated with baseline serum sclerostin in univariate and multivariate analysis
Venner et al. [21]	– Microarray analysis of kidney transplant biopsies performed to identify the changes in pure antibody-mediated rejection	– In 703 biopsies, 2603 transcripts were significantly changed in antibody-mediated rejection vs. all other biopsies – Sclerostin transcript decreased in biopsies of antibody-mediated rejection which reflect expression in the normal renal microcirculation and loss with endothelial dedifferentiation	NDA	NDA
Evenepoel et al. [12]	– 50 De novo KTRs, 23 KTRs undergoing parathyroidectomy and in 50 CKD patients are observed in a longitudinal cohort study for 1 year – Serum sclerostin was determined with an ELISA (TecoMedical)	– Median serum sclerostin levels decreased by 61.2% during the first 3 months after kidney transplantation (1.24 vs. 0.44 ng/mL, *P* < 0.0001) but increased between 3 and 12 months toward levels observed in CKD counterparts (0.63 ng/ml) – Increase of sclerostin between 3 and 12 months significantly correlated with the decrease of PTH in the same period – PTH independently associated with lower sclerostin both at time of kidney transplantation and at 1 year – Sclerostin significantly increased after parathyroidectomy (0.49 vs. 0.32 ng/ml) – Male sex, lower eGFR, and lower PTH were significantly associated with higher sclerostin which explain 38% of the variation of circulating sclerostin	– Lower eGFR was associated with higher sclerostin	NDA
Hsu et al. [11]	– In 68 KTRs, brachial-ankle PWV measured which greater than 14.0 m/s was determined as high arterial stiffness – Sclerostin levels were measured with an enzyme immunoassay (Biomedica)	– Mean sclerostin levels were 69.61 ± 26.04 pmol/l – Age, phosphorus, and log osteoprotegerin were positively associated with sclerostin – No significant correlation observed between sclerostin and Intact PTH	– eGFR was negatively correlated with sclerostin levels	30 KTRs (44.1%) with high arterial stiffness had higher sclerostin compared to KTRs with low arterial stiffness: 71.06 ± 23.52 vs. 50.57 ± 24.57 pmol/l – In multivariate analysis, sclerostin (OR:1.052, 95% CI 1.007–1.099) was independent predictor of arterial stiffness
Makówka et al. [36]	– In 35 KTRs, changes of mineral and bone biomarkers including sclerostin were measured in 9-month period – Urine sclerostin/creatinine ratio was assessed in parallel from month 1 after kidney transplantation – Serum sclerostin was determined with an ELISA (TecoMedical)	– Sclerostin levels were 1.95 ± 1.13, 1.51 ± 1.15, 1.2 ± 1.07, 0.84 ± 0.7, 0.99 ± 0.79, 1.05 ± 0.87, 1.24 ± 0.89, 0.85 ± 0.51, 0.92 ± 0.74, 1.35 ± 1.11 ng/ml, at Day 0, Week 1, Week 2, Month 1, Month 2, Month 3, Month 4, Month 5, Month 6, Month 9, respectively, showing 31% decrease from Days 0 at Month 9	– There was a significant increase in urinary elimination of sclerostin starting from the second month after transplantation	NDA
Tartaglione et al. [9]	– In 80 KTRs the relationship between serum sclerostin eGFR, calcium, phosphate, AP, iPTH, klotho, intact FGF-23, 25D and 1, 25D. 30 Healthy subjects served as control – Sclerostin levels were measured with sandwich ELISA kit (Biomedica)	– Sclerostin not different in KTRs and controls (23.7 pmol/l vs. 26.6 pmol/l, respectively) – Sclerostin correlated negatively with AP and positively with FGF-23 and 25D suggesting anti-anabolic role and inhibitory action on vitamin D of sclerostin action – AP and 1, 25D were negative and 25D and Klotho were positive predictors of sclerostin	– No correlation was found sclerostin and eGFR and CKD stages	NDA
Hernandez et al. [37]	– 29 KTRs randomized to zoledronic acid (5 mg, iv at the time of kidney transplantation) plus cholecalciferol (25,000 UI every 15 days for 12 months) for 12 months (*N* 15), vs. only cholecalciferol at the same dose (*N* 14) – Bone biopsies were performed at baseline (at the time of kidney transplantation) and after 12 months of treatment. control group comprising 29 age- and gender-matched iliac crest bone samples – Histomorphometric evaluation was performed in bone and bone marrow adipocytes – Sclerostin expression in osteocytes was evaluated by immunohistochemistry – Serum sclerostin was determined by ELISA (TecoMedical)	– Sclerostin levels were not decreased after kidney transplantation – Osteocyte sclerostin expression after kidney transplantation decreased with the use of ZA compared to KTRs who only taking cholecalciferol – Kidney transplantation fails to normalize bone marrow adiposity which are higher in kidney transplantation vs. healthy controls and even gets worse with the use of zoledronic acid – There was inverse correlation between osteocyte sclerostin expression and bone marrow adiposity	NDA	NDA
Basir et al. [10]	– In 78 KTRs, serum sclerostin measured and lumbar and femoral neck bone mineral densities and *T* and *Z* scores were measured by DEXA in a cross-sectional study – Patients with parathyroid adenoma or parathyroidectomy history were excluded – Sclerostin measured by ELISA kit (Elabscience, Houston, TX, USA) on an enzyme-linked immunosorbent assay device (Thermo Fisher Scientific)	– The osteoporotic group had lower sclerostin vs. non-osteoporotic group (405.9 vs. 521.7 ng/dl) – SCL not correlated with cumulative corticosteroid dose, intact PTH, bone mineral density, and *T* scores	– No relationship between eGFR and sclerostin	NDA
Evenepoel et al. [24]	– In 518 adult KTRs bone mineral density measured at several skeletal sites within 14 days post-transplant and bone turnover markers are measured – Serum sclerostin was determined with an ELISA (TecoMedical)	– Baseline sclerostin levels were 2.21, 1.84, and 1.79 ng/l in normal, osteopenic, and osteoporotic patients (*P* 0.01) – Baseline sclerostin was positively associated with FGF-23 and negatively with PTH and 1, 25D – Baseline sclerostin levels with and without incident fracture were not different 2.08 ng/l [1.64–2.48] vs. 1.87 ng/l [1.30–2.67]	NDA	NDA
Marques et al. [25]	– In an open label, randomized trial, 17 KTRs assigned to Zoledronate (*N* 17) and control (*N* 17) before kidney transplantation – Bone biopsy performed at the time of kidney transplantation and after 12 months to analyze bone histomorphometry – DXA and high-resolution peripheral quantitative computed tomography performed in zolendronate group (*N* 16) and in controls (*N* 16) at the end of study – Serum sclerostin was measured using ELISA (TecoMedical)	– Serum sclerostin decreased after kidney transplantation (1.05 to 0.52 ng/ml) but no effect of Zoledronate was found on this parameter – No significant correlations were seen between changes in serum sclerostin and changes in DXA, tomography bone histomorphometry	NDA	NDA
Araújo et al. [38]	– 31 Bone biopsy specimens obtained in KTRs who randomized to control or zoledronate groups for 1 year but data were shown for all patients – Serum sclerostin was measured using ELISA (TecoMedical)	– Sclerostin decreased after kidney transplantation – No change in bone volume or mineralization occurred after kidney transplantation, however low bone turnover became more frequent after kidney transplantation ranging from 10 (32%) to 20 (65%) – Bone expression of sclerostin and bone sclerostin increased, whereas serum sclerostin decreased after kidney transplantation suggesting sclerostin decrease is not due to a decrease in the bone osteocytic synthesis but due to improved renal function	NDA	NDA
Coban and Okten [19]	– The correlation between sclerostin and bone mineral density investigated in KTRs with more than 1 year after kidney transplantation – 80 KTRs and 40 healthy controls included – Serum sclerostin measured with (ELISA, Elabscience, CA, USA)	– There was no difference in sclerostin levels between KTRs and healthy individuals – High sclerostin associated with lower proximal femur bone mineral density	– No significant correlation between sclerostin and eGFR	NDA
Evenepoel et al. [39]	– 69 KTRs with steroid minimization immunosuppressive protocol underwent into a 5-year prospective observational study to evaluate changes in bone mineral density, mineral metabolism, and bone remodeling at baseline, 1 year after kidney transplantation and 5 year after kidney transplantation – Serum sclerostin was measured using ELISA (TecoMedical)	– Sclerostin significantly decreased 67.4% at first year after kidney transplantation from 2.217 to 0.74 ng/l but increased significantly at post-transplant 5 years to 0.88 – In femoral neck bone mineral density, as compared with losers, gainers were characterized by higher sclerostin 2.53 vs. 1.82 ng/l at 1 year but not different at 5 year	NDA	NDA
Zeng et al. [18]	– 600 stable KTRs were followed for 3 years which end-point is all-cause mortality – Baseline blood and urine samples including sclerostin measured for analysis and clinical data were collected at study entry – Sclerostin was measured with the use of a ELISA (Biomedica)	– Non-survivors (*n* 65) had higher plasma sclerostin levels than survivors (*N* 535) (57.31 ± 30.28 pmol/l vs. 47.52 ± 24.87 pmol/l)	– In subgroup analysis sclerostin is an important all-cause mortality risk factor among patients with lower eGFR (< 44.41 ml/min/1.73 m^2^)	– In multiple Cox regression analysis, sclerostin was independent predictor of all-cause mortality (HR 1.011; 95% CI 1.002–1.020)
Ferreira et al. [20]	– In 67 KTRs, sclerostin, densitometry (by DXA), bone histomorphometry (by biopsy), and CAC (by cardiac CT) performed 1 year after kidney transplantation – Sclerostin was measured with the use of a ELISA (Biomedica)	– Sclerostin after in year kidney transplantation was 0.7 (0.49–0.96) ng/ml – High sclerostin associated with high bone mineral density both at spine and femur	NDA	NDA
Ferreira et al. [14]	– 84 KTRs prospectively followed for 12 months – Demographic, clinical, and echocardiographic data were collected, laboratory evaluation, bone biopsy, and X-ray of the pelvis and hands were performed – Patient and graft survival were recorded – Vascular calcifications measured by through X-ray of the pelvis and hands by Adragão score – Sclerostin was measured with the use of a ELISA (Biomedica)	– Baseline sclerostin levels were elevated 1.9 (1.2–2.8) ng/ml – Low bone turnover was associated with high levels of sclerostin – Sclerostin levels were 2.2, 1.3, and 1.1 in low, normal, and high turnover bone disease – Interdependently, high bone turnover was independently associated with lower sclerostin levels – Isolated low bone volume was associated with higher levels of sclerostin (2.8 vs. 1.9 ng/Ml) – Bone mineralization was not associated with sclerostin	NDA	– In KTRs with mild score of Adragao vascular calcification score had lower sclerostin vs. higher scores 1.89 ± 0.9 vs. 2.45 ± 0.9 ng/ml suggesting high sclerostin was associated with vascular calcification – Sclerostin was the only predictor of survival with higher levels had higher death (HR = 3.24, *P* = 0.041 – Sclerostin levels were not different between KTR with or without heart valve calcification (2.1 vs. 1.9 ng/ml)
Ferreira et al. [22]	– In 69 KTRs pre- and 1-year post-kidney transplantation clinical, biochemical, histological (bone biopsy), and imaging (echocardiography radiography of the pelvis and hands) parameters measured – Sclerostin was measured with the use of a ELISA (Biomedica)	– Sclerostin decreased in all KTRs after kidney transplantation (from 1.9 [1.3–2.7] ng/ml to 0.7 [0.5–1.0] ng/ml – The impact of sclerostin was assessed on three bone associated changes namely: bone remodeling, bone volume, and bone mineralization – In KTRs with decreased bone remodeling after kidney transplantation, sclerostin levels lower at 1 year after kidney transplantation vs. baseline (0.5 [0.3–0.9] ng/ml vs. 0.8 [0.6–1.0] ng/ml and a higher percentage reduction in sclerostin (68.9% versus 59.4%) – In KTRs with bone volume loss, baseline sclerostin levels higher compared to KTRs without bone loss (2.5 [2.2–4] ng/ml vs. 1.7 [1.2–2.7] and a significant decrease and in sclerostin values from baseline (− 2.2 [− 3.1 to − 1.2] vs. − 1.1 [− 1.6 to 0.7] – The decrease in bone volume after the kidney transplantation was associated with the highest levels of sclerostin at baseline – Mineralization was not effected by sclerostin after kidney transplantation	NDA	– Sclerostin levels higher in KTRs with higher CAC (by Agatston score) – Baseline sclerostin in Agatston percentile ≤ 50%, 51–90%, and > 90% were 1.7, 2.1, and 2.2 (ng/ml) but not different at 1 year (0.6, 0.7, and 0.9 ng/Ml, respectively) – In the multivariate analysis, baseline sclerostin was positively associated with CAC percentiles
Magalhães et al. [13]	– In 13 KTRs, the relationship between histomorphometric analysis with serum biomarkers analyzed prospectively – Plasma sclerostin measured by ELISA (Biomedica)	– Non-significant decrease of sclerostin at 12 months vs. baseline observed 1502 ± 201.4 pg/ml vs. 1307 ± 143.5 pg/ml – At 18 months post-kidney transplantation sclerostin levels began to increase (1544 ± 287.8 pg/ml), being significantly higher at 24 months after kidney transplantation in comparison with the levels observed at 12 months (1307 ± 143.5 pg/ml at 12M vs. 2094 ± 318.6 pg/ml at 24M) after kidney transplantation – Sclerostin did not correlate with calcium, phosphorus, vitamin D, PTH, FGF-23 - Sclerostin correlated significantly and positively with trabecular separation and negatively with trabecular number independent of calcium, phosphorus, intact PTH and plasma creatinine., both indicating that increased levels of this new biomarker is related to low bone volume	NDA	NDA
Koh et al. [17]	– In 591 KTRs, sclerostin measured before kidney transplantation and followed prospectively for 5 years – Abdominal aortic calcification and brachial-ankle PWV measured at pre-transplant screening and 3 and 5 years after kidney transplantation – Method for sclerostin measurement: Not reported	– The median sclerostin level was 296.6 (226.8–404.5) pg/ml – Serum PTH were inversely associated with sclerostin, whereas serum calcium, 25-D, and 1, 25-D levels were positively associated with sclerostin	– The rejection was higher in the group with low sclerostin only for acute antibody-mediated rejection	– In linear regression analysis, higher sclerostin levels were associated with faster increase in post-transplant abdominal aortic calcification and PWV – In multivariable analyses, 1% increase in sclerostin (98.1 [95% CI 18.8–177.3]) and the third tertile sclerostin levels (126.7 [95% CI 35.6–217.8]) were significantly associated with an increase in aortic PWV after KT – Stroke events showed an increasing pattern of 0.5%, 1%, and 3% as the sclerostin levels increased from the first to the third tertile
Wang et al. [15]	– In 79 KTRs, circulating sclerostin, DKK1, FGF-23 and α-klotho arterial stiffness (carotid-femoral PWV), carotid-radial PWV, augmented index and bone parameters were assessed before (M0), and at 3 (M3) and 6 months (M6) after kidney transplantation	– Sclerostin at MO, M3, and M6 were (2.06 ± 1.18, 0.81 ± 0.24, and 0.88 ± 0.29 ng/ml, respectively – The levels of sclerostin decreased at M3 and M6 vs. MO (not different between M3 vs. M6)	NDA	– Sclerostin positively associated with carotid-femoral and carotid-radial PWV throughout the 6 months of follow-up – The decrease of sclerostin and FGF-23 at M3 and M6 was associated with the decrease of cf-PWV and cr-PWV
Zuo et al. [60]	– In 531 stable KTRs the relationship between 25-D, 1, 25-D and iPTH, oxPTH n-oxPTH and sclerostin investigated – Sclerostin measured by ELISA (TecoMedical)	– Sclerostin levels were 50.61 ± 24.16 pg/ml – Sclerostin levels negatively associated with 1,25-D but not with 25-D – In multivariate analysis no associations between 25-D and 1, 25D were observed	NDA	NDA
Vigil et al. [113]	– Bone metabolism proteins including sclerostin measured in plasma in KTRs (*n* 57), hemodialysis patients (*n* 26), and healthy controls (*n* 31) – Sclerostin measured by a Luminex-based microbead assay using a MAGPIX microsphere analyzer	– Sclerostin levels were lower in KTRs vs. HD and control groups and no difference between control and HD – In KTRs, sclerostin is positively correlated with DKK1 and osteoprotegerin and FGF-23	NDA	NDA

*KTRs* kidney transplant recipients, *GFR* glomerular filtration rate, *NDA* no data available, *CKD* chronic kidney disease, *DKK1* Dickkopf-related protein 1, *iPTH* intact parathyroid hormone, *PWV* pulse wave velocity, *CAC* coronary artery calcification, *AP* Alkaline Phosphatase, *FGF-23* Fibroblast Growth Factor 23, *25D* 25-hydroxyvitamin, *1, 25D* 1, 25-dihydroxyvitamin D

### Relationship Between Sclerostin and Bone Parameters

We identified articles showing associations of sclerostin with PTH, fibroblast growth factor 23 (FGF-23), vitamin D, and klotho levels. With regard to PTH, sclerostin was shown to be either negatively associated with PTH [12, 17] or showed no association [13]. In general, sclerostin was positively associated with FGF-23 [9, 24]. The relationship between sclerostin and vitamin D is more complex. Sclerostin was correlated positively with 25 hydroxy vitamin D (25D) levels [9, 17] but correlated both positively [17] and negatively [9] with 1–25 hydroxy vitamin D levels (1-25D). Sclerostin was also positively correlated with klotho [9].

### Relationship Between Sclerostin and Bone Mineralization Bone Turnover and Bone Volume

In KTRs, there are various studies investigating the relationship of sclerostin with bone mineralization, bone turnover, and bone volume. With regard to bone mineralization, some studies have shown that lower sclerostin levels were associated with lower bone mineral density [10, 20, 24] while other showed that high sclerostin was associated with low bone mineralization [19] and others showed no association [25]. Some studies documented associations of high sclerostin with low bone turnover [20]. Various studies have also shown associations of elevated sclerostin with low bone volume in KTRs [13, 14, 20, 22].

### Relationship Between Sclerostin and Graft Function

With regard to graft function, some studies have shown negative associations between sclerostin and glomerular filtration rate, GFR [11, 12]. While others showed no association [9, 10, 23]. We did not identify any studies investigating the relationship between protein–albumin/creatinine ratio and sclerostin in KTR.

### Relationship Between Sclerostin and Vascular Calcification and Cardiovascular Outcome

The studies regarding sclerostin and vascular calcification in KTRs were also inconsistent with some studies showing positive associations [14, 17, 22] while others studies showing negative associations [16]. Sclerostin has been also associated positively with arterial stiffness [11, 15, 17]. However, we did not find any study investigating the relationship between sclerostin and hard cardiovascular end points such as myocardial ınfarction, heart failure, stroke, and peripheral arterial disease in KTRs. Lastly, there are also studies showing that high sclerostin was associated with lower antibody-mediated allograft rejection [21] but higher mortality [18].

## Discussion

Although kidney transplantation is the preferred treatment choice for end-stage kidney disease, long-term prognosis is hampered by chronic allograft failure due to many reasons and associated with long-term cardiovascular morbidity and mortality [2]. Thus, there is unmet need for new biomarkers to early detect and manage kidney dysfunction and cardiovascular events in KTRs. One of these potential but less studied marker in sclerostin in KTRs. As identified in this review, sclerostin was associated with laboratory, clinical, and bone-related parameters in KTRs with conflicting results. To frame these results, we briefly review current knowledge of physiology of sclerostin, then summarize sclerostin changes before and after kidney transplantation and its association with GFR. We also shed light regarding the association between sclerostin bone parameters, vascular calcification, and arterial stiffness. Lastly, we defined conflicting issues, and potential drawbacks of sclerostin measurement.

### Sclerostin Physiology

Sclerostin is mainly synthetized in the bone by osteocytes and to a lesser extent by osteoclast precursors. The main action of sclerostin is to inhibit bone formation and mineralization by blocking osteoblast differentiation and function. At molecular level, the action of sclerostin is through wingless-related integration site (Wnt) signaling in the bone. Sclerostin binds to low density lipoprotein receptor-related proteins on cells of the osteocytes and inhibit the activity of Wnt/ß-catenin canonical signaling pathway. Sclerostin is one of the Wnt inhibitors blocks the extracellular binding of Wnt to transmembrane receptors complex thus preventing translocation beta-catenin to nucleus and subsequent gene expression. In murine knockout models, deficiency of sclerostin associated with high bone mass [26]. Sclerostin also impacts osteoblast maturation and decreases expression of osteoprotegerin (OPG) which promotes bone formation by mature osteoblasts. Thus sclerostin results in the decreased OPG and OPG/RANKL (the receptor activator of nuclear factor kappa-B ligand) ratio, which increases osteoclastic bone resorption [9, 27–29]. Mechanical stimulus or loading is also important for sclerostin synthesis which reduce osteocyte sclerostin generation. Reference [30] explaining the lowering impact of physical activity on sclerostin levels [31]. Reciprocally, in animal models, with unloaded bone, sclerostin levels increase dramatically. Furthermore, injection of sclerostin blocking antibodies in rats increases trabecular and cortical bone mass and induced a strong increase in bone formation rate [32]. A part from actions on bone, sclerostin has metabolic effects and higher sclerostin was associated with higher body mass index, fat mass, and insulin resistance [33]. Furthermore, sclerostin is thought to play a role in vascular calcification [34] and arterial stiffness [35] as discussed further below.

### Sclerostin Levels, Kidney Transplantation, and Graft Function

There are various studies that measure sclerostin levels only before kidney transplantation (baseline) and only after kidney transplantation and both before and after kidney transplantation to catch up longitudinal changes. We found some studies showing that sclerostin decreased after kidney transplantation in the first weeks but then start to increase beginning at third month [12] or sixth month [23]. Other studies showed fluctuating sclerostin levels after kidney transplantation [15, 36]. In general, sclerostin appears to decrease after kidney transplantation [22, 25, 37–39] but there are also studies showing no difference of sclerostin before and after kidney transplantation [13]. Even in the same study, it was shown that despite increasing bone synthesis of sclerostin, circulating sclerostin has found to be decreased [38] suggesting that serum sclerostin does not necessarily reflect sclerostin levels in bone [40].

In this review, we found that sclerostin was either negatively associated with GFR or no association was found. In general, longitudinal studies measuring multiple sclerostin showed that sclerostin decreased in early period but increased thereafter beginning at month 3 [12], month 6 [23], and month 12 [13]. Why does this dynamic changes occur? Although the current answers are not known, some speculations can be made. First, sclerostin having a low molecular mass and positive charge can be filtered through glomerulus and in physiological conditions, most of this filtered sclerostin is reabsorbed in the proximal tubule [41]. However, in the early post-transplant period, proximal tubular dysfunction—which is most commonly related to ischemia–reperfusion injury—may induce overloading proteinuria resulting in the losses of substantial amounts of sclerostin in the urine resulting low plasma sclerostin [12]. Second, as sclerostin levels increase as GFR declines [42], it is possible that GFR restoration after kidney transplantation may be associated with lower sclerostin. However, others suggest that despite reductions in GFR, renal fractional excretion of sclerostin may increase up to 20 times and only moderate elevations of sclerostin occurs [43]. Third, despite lowering of PTH levels after kidney transplantation, this may take time and high PTH levels during early post-transplantation period may contribute to low sclerostin due to inhibitory effect of PTH on sclerostin [12, 44]. Fourth, high glucocorticoid use in early post-transplant period may suppress serum sclerostin levels, although prolonged treatment appears to be associated with increased serum sclerostin [45]. However, this issue is complex since in vitro studies showed that glucocorticoid administration to osteoblasts increased sclerostin production but mice treated with glucocorticoids exhibited decreased sclerostin expression [46]. In humans, the acute administration of glucocorticoids over a 96-h period induced a decrease in serum sclerostin, whereas long-term administration was associated with increased sclerostin levels at 12 months after therapy initiation in a dose-dependent fashion [47]. It was also suggested that the discordance between serum and bone sclerostin may be caused by glucocorticoid treatment [48, 49]. Fifth, the increase in physical activity in these patients after kidney transplantation may also help explain decrease of sclerostin [50]. Sixth, bone loss, which commonly occurs early after kidney transplantation [51] may be associated with low sclerostin.

**Fig. 2:**
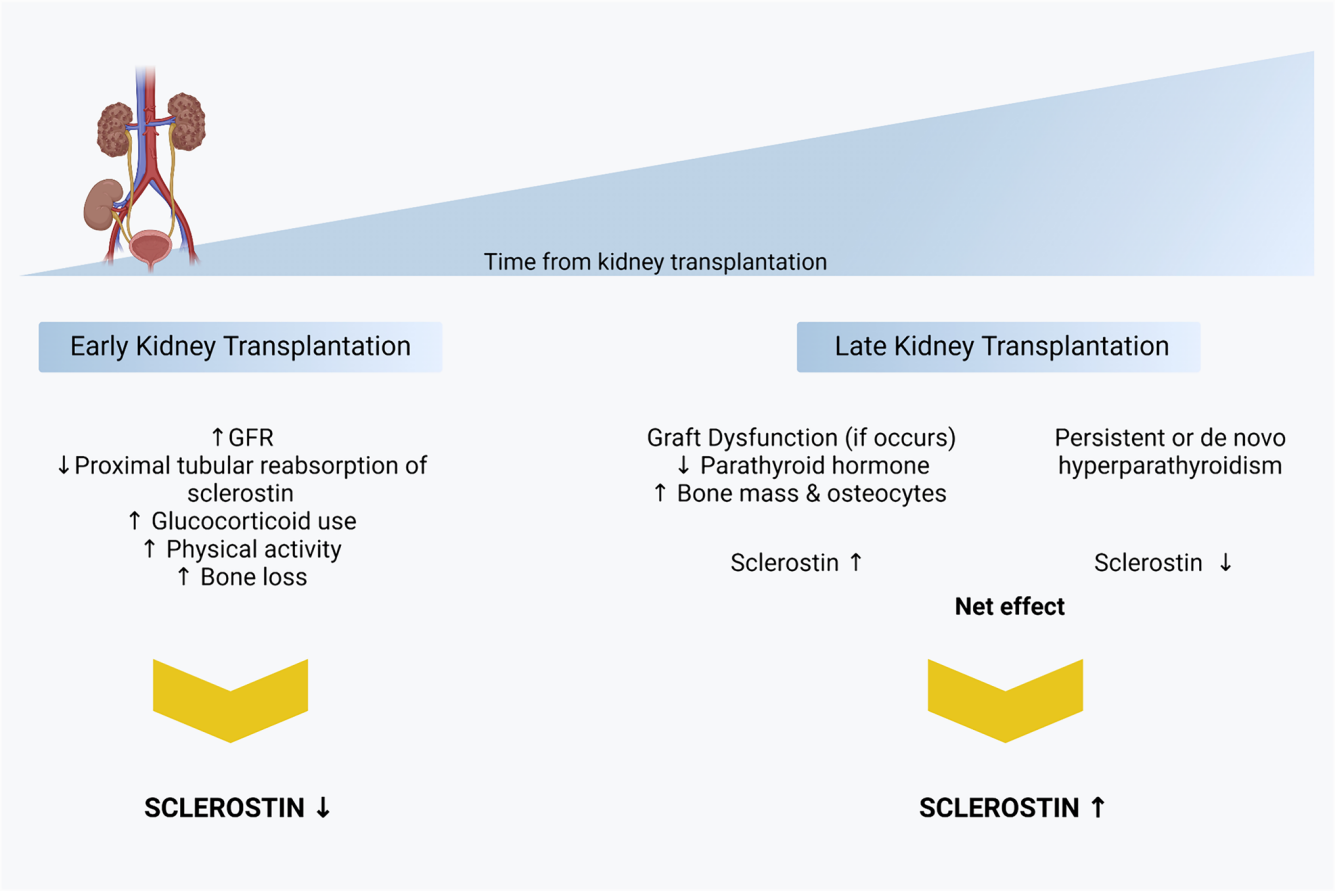
The dynamic changes of sclerostin after kidney transplantation. It is generally shown that, circulating sclerostin levels decrease after early kidney transplantation but then rebound increase occurred as the duration of kidney transplantation increased although the elevation of sclerostin after KT occurred at different intervals beginning from 3 to 12 months. Even though the exact mechanisms of this dynamic changes are unclear, potentially different factors impact circulating sclerostin levels at the early and late phase after KT

Although all these hypotheses explain the reduction of sclerostin in the early post-transplant period, there is no satisfactory explanation for increase of sclerostin in the long-term period identified in some studies [13, 23]. One can assume that these changes are closely associated with changes in GFR but this is not the real case. For example, although Evenepoel et al. showed inverse association between sclerostin and GFR [12] Bonani et al. showed no association between GFR and sclerostin [23]. Thus, although GFR may impact the sclerostin levels; it should not be the only reason for elevation of sclerostin after kidney transplantation. Another alternative hypothesis is reduction of PTH after kidney transplantation in long term. As PTH levels inhibit sclerostin under physiologic conditions, lack of PTH inhibition potentially elevates sclerostin in non-early period of kidney transplantation. Lastly, as high sclerostin levels also reflect increased osteocyte number and skeletal mass [52, 53]; the increase in sclerostin after kidney transplantation may simply reflect increased bone mass after kidney transplantation. Indeed, although bone loss and fractures are common in the early post-kidney transplantation period, in long-term kidney transplantation patients bone mineral density improves progressively as time after kidney transplantation increased [54]. Based on these findings, the sclerostin trajectories after kidney transplantation are not uniform and complex and multiple etiologies play a role for final sclerostin levels (Fig. 2). Again, it is of note that circulating sclerostin levels may not reflect bone sclerostin at the site of clinical important action.

### Association of Sclerostin with Bone Parameters, Bone Histomorphometry, Bone Mass, Bone Mineralization, and Kidney Transplantation

The MBD pathology is very complex, involving various parameters such as bone-related clinical parameters (calcium, phosphorus, PTH, vitamin D, bone-specific alkaline phosphatase FGF-23, klotho), bone mineralization, bone turnover, and bone mass. In kidney transplantation, there are no specific studies investigating the reciprocal changes between calcium-sclerostin and phosphorus-SCL. The relationship between sclerostin and phosphorus was a complex issue and not fully elucidated. While sclerostin levels were associated with phosphorus levels in hemodialysis patients [55], a recent study demonstrated that a treatment with an anti-sclerostin antibody reduced serum levels of intact FGF-23 and increased serum phosphate levels [56]. This difference may be due to fact that in hemodialysis patients despite increased FGF-23 levels phosphate cannot be excreted due to oliguria/anuria. Furthermore, although the regulation of FGF-23 by sclerostin is suggested to be indirect and mediated by the control of bone turnover [57], a cell study using the osteocytic cell line IDG-SW3 suggested the direct stimulating effects of sclerostin on the synthesis of FGF-23 [58]. These issues are not studies in KTRs and subject to future investigation.

With regard to PTH, sclerostin was shown to be either negatively associated with PTH [12, 17] or showed no association [13]. The relationship between sclerostin and PTH seems to be dependent on pattern of PTH secretion. For example, circadian but not continuous increases in PTH have anabolic effects on bone partly due to activation of osteocyte PTH receptors which downregulate sclerostin expression [59]. Therefore, anabolic effects caused by a reduction in sclerostin may only occur by intermittent rise in PTH. Sclerostin was correlated positively with 25D [9, 17] and correlated either positively [17] or negatively [9, 60] with 1-25D. These findings may be explained by the interdependent relationship between vitamin D and sclerostin. Higher circulating 25D by resulting in higher intracellular 1, 25D which in turn, favor sclerostin synthesis by osteocytes. However, increased sclerostin levels exert a negative feed-back on 1, 25D synthesis [9]. Studies are also present showing that sclerostin was positively correlated with klotho [9] and with FGF-23 [9, 24] Indeed, it is known that sclerostin increases FGF-23 by inhibiting PHEX (phosphate regulating endopeptidase X-linked) [61] which stimulates FGF-23 degradation [62] thus reducing its activity.

**Fig. 3:**
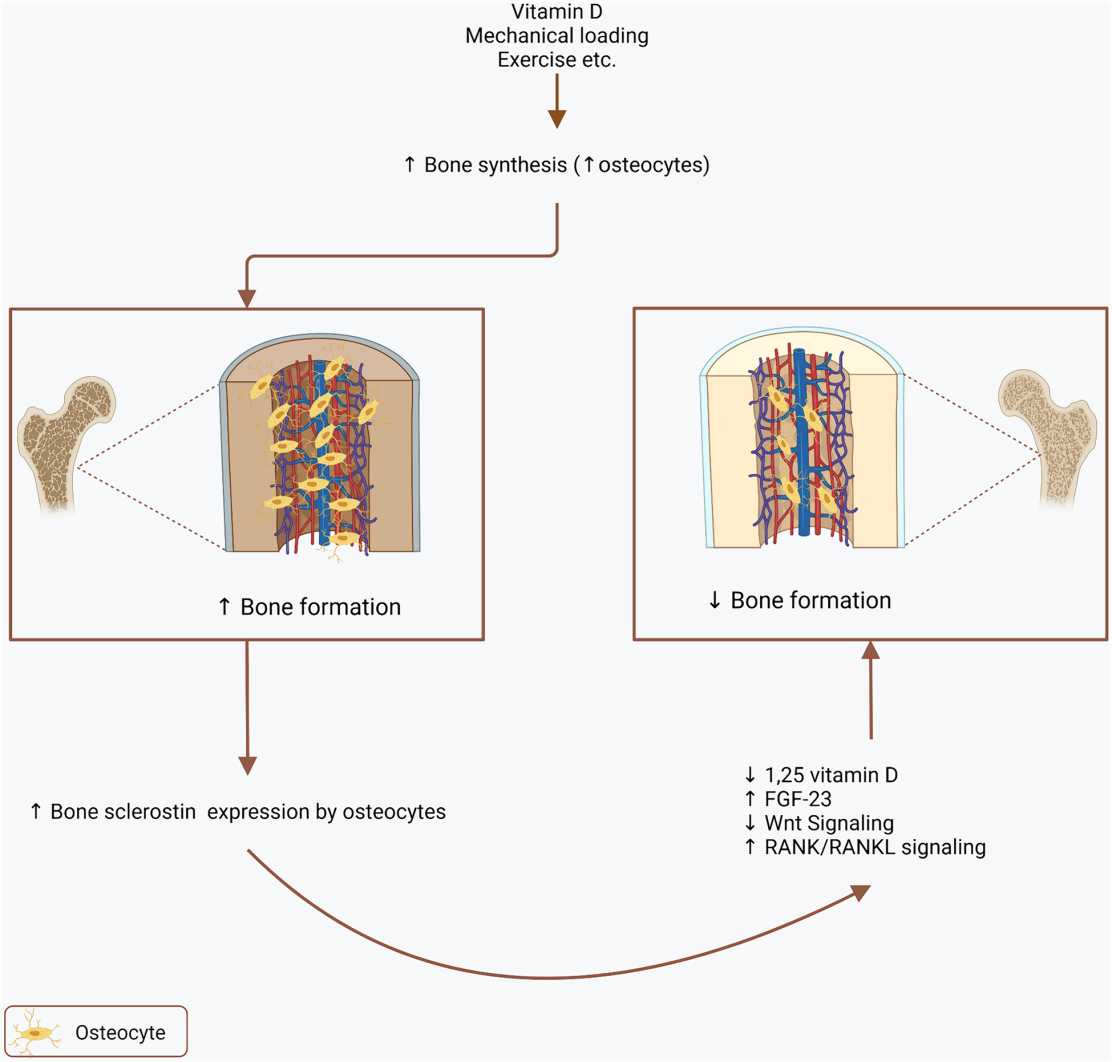
Relationship between sclerostin and bone. When bone synthesis increases, expression of bone sclerostin increases by expanding osteocyte number. Sclerostin in turn decrease 1, 25-dihydroxyvitamin D and wingless-related Wnt). Simultaneously, increased sclerostin also increase FGF-23 and also (RANK/RANKL signaling which promotes bone resorption as a negative feedback to normalize increased bone formation. *Wnt* wingless-related integration site, *FGF-23* Fibroblast Growth Factor 23, *RANK* receptor activator of NF-Κb, *RANKL* Ligand for RANK

With regard to association of bone volume, bone mineralization, and bone turnover with sclerostin, there were diverse and contrasting findings identified in the current review. The reasons for this discordant findings are not known but there are two opposite opinions: The first is compatible with the normal physiologic function of sclerostin which is to inhibit bone formation by blocking Wnt signaling and bone formation [63]. Other opinion suggest that high sclerostin levels reflect increased osteocyte number and increased skeletal mass [52, 53]. To combine these two opinions one may hypothesize that there may be a feedback regulation during bone hemostasis that is when bone formation increased, sclerostin also increased which then decrease bone formation by suppressing Wnt signaling and normalizing increased bone formation (Fig. 3). Whether this hypothesis holds true especially in kidney transplantation needs further investigation. However, it is clear that romosozumab—a humanized monoclonal antibody sclerostin inhibitor- increased bone mass in healthy men and postmenopausal women with low bone mass [64]. Furthermore, higher levels of sclerostin was associated with increased fracture risk [65, 66]. It was also shown that postmenopausal women with osteoporosis, romosozumab was associated with a lower risk of vertebral fracture than placebo [67]. In patients receiving hemodialysis treatment romosozumab increased bone mineral density without increased cardiovascular events [68]. However, it was suggested that romosozumab may be associated with increased cardiovascular events and should not be used in patients with a recent cardiovascular event and should be used cautiously in patients with high cardiovascular risk [69]. To date there is no study investigating the role of romosozumab in KTRs.

### Sclerostin Vascular Calcification, Arterial Stiffness, and Kidney Transplantation

Vascular calcifications (VC) are highly prevalent in KTRs which are associated with an increased risk of cardiovascular events and long-term mortality [70]. Vascular calcification is not passive but an active process which shares many features with bone formation [71]. During VC, a complex interplay of variety of molecules and signaling pathways occurs. The small integrin-binding ligand N-linked glycoprotein (SIBLING) family proteins including osteopontin, bone sialoprotein, [72, 73], osteoprotegerin [74], high phosphate and low pyrophosphate [75] all play role. Among signaling pathways bone morphogenetic protein and Runt‐related transcription factor 2 signaling, AMP‐activated protein kinase signaling pathway, The phosphatidylinositol 3‐kinase (PI3K)-AKT signaling pathway, NOTCH signaling pathway, Wnt-β-catenin signaling all play a role for VC [76]. Among these processes the role of sclerostin in VC is mostly associated with Wnt-β-catenin signaling. During VC, vascular smooth muscle cells undergo osteogenic dedifferentiation, which goes along with increased activation of the Wnt/β-catenin signaling cascade [77]. Overall, in the vessel wall, Wnt-β-catenin signaling promotes atherosclerosis and VC. However, in contrast, expression of Wnt antagonists in calcifying vascular smooth muscle cells increases which include sclerostin [78, 79]. An in vitro study showed that vascular smooth muscle cells undergo terminal trans-differentiation into an osteocyte-like cell type with sclerostin expression during calcification [78] suggesting that sclerostin actively play a role in this process. Additionally, the elevated sclerostin expression in calcifying aortic tissue was found in an in vitro model of uremic vasculopathy [80]. During in vivo models of acute kidney injury [81] and CKD [82] vascular (aortic) expression of sclerostin was also increased. This evidence suggests that increased sclerostin may be involved in development of VC and arterial stiffness.

Studies in KTRs demonstrated that sclerostin was positively associated with VC [11, 14, 15, 17, 22]. On the contrary, other suggest that sclerostin does not have facilitative role in the VC but has a protective role and sclerostin expression represents a counter regulatory mechanism aimed to suppress the progression of VC. This also aligns with the physiological role of sclerostin that is, the downregulation of matrix mineralization. In vitro studies also showed that sclerostin was able to suppress vascular smooth muscle cell osteoblastic differentiation [83, 84]. Based in this opinion, sclerostin produced in the calcified vessels protects vessel wall from further calcification and the produced sclerostin by vessel wall may spill-over to the serum compartment, leading to increased serum sclerostin levels in patients or animals with vascular media calcification [85, 86]. Thus non-skeletal (vascular) production of sclerostin in fact reflect a protective mechanisms against VC which contribute to the high circulating sclerostin levels in patients with advanced CKD [87]. To further ratify this hypothesis, in CKD-induced VC sclerostin knock-out mice showed more VC in cardiac vessels. Moreover, same study showed that in warfarin induced VC without CKD, DBA/2J mice showed more vascular calcification with anti-sclerostin antibody treatment suggesting a protective effect of sclerostin during VC [88].

**Fig. 4:**
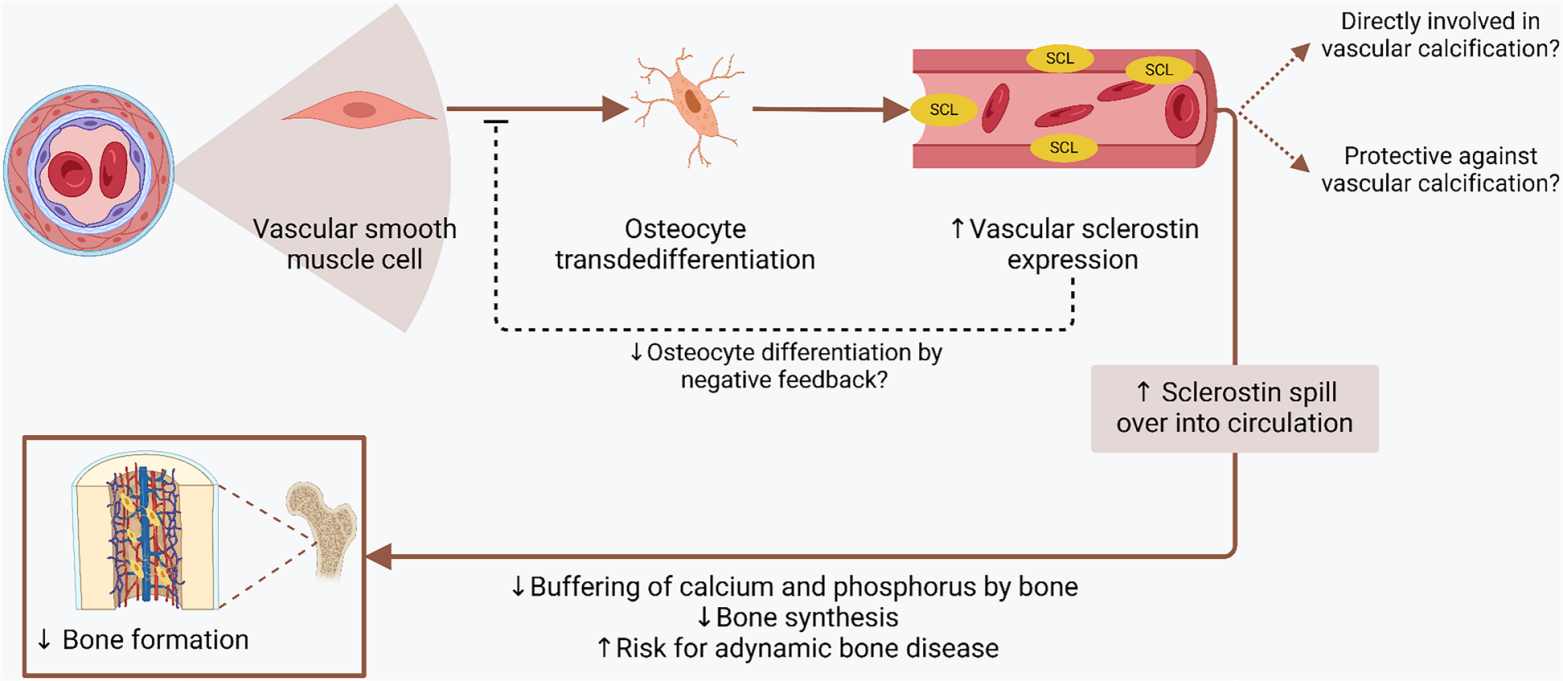
Sclerostin and vascular calcification. Vascular calcification is an active process which involves the transdedifferentiation of vascular smooth muscle cells into osteocyte-like cells. These differentiated cells secrete sclerostin. The literature regarding vascular calcification is conflicting. It was found that sclerostin is associated with positively and actively in vascular calcification process. On the other hand others proposed that sclerostin is protective against vascular calcification and the increased sclerostin supress vascular smooth muscle transdedifferentiation into osteocytes thus decreasing vascular calcification as a negative feedback. Although which hypothesis is true is not known currently, both of them suggest that increased bone sclerostin expression occurs during vascular calcification which spill over to circulation and effect bone metabolisms as a bone–vascular cross-talk

It is of note that a bone biopsy study showed that increased circulating sclerostin levels in the elderly were not accompanied by an increase in sclerostin mRNA levels in bone, fueling the hypothesis that the contribution of non-skeletal sources, including the vasculature, to circulating levels may be relevant [89]. In KTRs, a study showed that baseline sclerostin level was inversely associated with progression of abdominal aortic and coronary artery calcifications in a multivariable model but after adjusting for baseline calcification score, the significance was lost [16]. Thus it is important to determine whether systemic levels of sclerostin play a causal role in the development of vascular calcifications or whether sclerostin production simply reflects phenotypic changes in the vessel walls as a protective negative feedback (Fig. 4). This is important issue, since if the second opinion is true and if sclerostin was actively inhibiting VC, the inhibition of sclerostin would be detrimental by promoting further VC in the vessel wall [26].

This calcification paradox is also important when sclerostin produced from the calcified vasculature spills into the circulation, it inhibits bone formation and consequently inhibits the ability of the bone to buffer calcium and phosphorus, allowing them to be deposited on the vascular walls [17]. Indeed, a recent study showed that when uremic aorta from a rat with CKD was transplanted into an animal with healthy kidney, the bone mineral density of the (otherwise healthy) recipient animal decreased, by impaired mineralization of bone. Additionally, the expression of sclerostin in bone increased in the recipient animals [90]. This study confirms the notion that bone-vascular axis in CKD is bidirectional and is not a “one-way route.” In KTRs, some studies have also shown that arterial stiffness was associated with arterial stiffness measured by pulse wave velocity, PWV [11, 17]. As PWV is also a strong predictor of cardiovascular events and all-cause mortality in transplant patients [91, 92], sclerostin levels before kidney transplantation would help with risk stratification by predicting deterioration of arterial stiffness after kidney transplantation [93, 94]. Future studies are needed to determine whether associations with surrogate measures such as vascular stiffness translate to differences in clinically important cardiovascular outcomes.

## Areas of Uncertainty

The relationship with sclerostin, and graft function, bone and VC is not only complex but also conflicting in KTRs. One of the potential reasons for conflicting findings is the heterogeneity of studies with respect to sclerostin measurement (baseline, after or longitudinal measurement of sclerostin), population characteristics, glucocorticoid exposure etc. These conflicting data may be explained by case-mix, and different factors used in multivariate analysis. Moreover, the relationship may not be linear, but U-shaped [79]. Second, the difference between sclerostin assays may be of importance. Although commercially available sclerostin assays show good correlations between each other, they demonstrate significantly different results in terms of absolute sclerostin concentrations in serum. Therefore, studies using different sclerostin assays cannot be compared directly [50, 95, 96]. Delanaye et al. compared four different assays for measurement of sclerostin namely Biomedica, TecoMedical, R&D Meso Scale Discovery in 39 prevalent dialysis patients and 82 non-dialysis patients. observed large differences in median sclerostin concentrations (in pg/ml) and the concordance correlation coefficient between assays was poor. Sclerostin measured by Biomedica and TecoMedical, were negatively correlated with GFR which is not observed when sclerostin measured by Meso Scale Discovery and R&D. The associations between sclerostin and gender age, or parathormone were also different according to the assay used [97]. This is a clinically important limitation in comparing studies and it will be probably more important in the setting of kidney transplantation which GFR can fluctuate.

The relationship between sclerostin and mortality in kidney transplantation also deserves mention. Studies evaluating the association between circulating sclerostin levels and mortality in CKD have yielded inconsistent results, with some investigators reporting high circulating sclerostin levels to associate with better survival [85, 98] and other investigators reporting an inverse [99] or no association [100, 101]. In KTRs, one study reported that sclerostin was positively associated with mortality in KTRs [18]. More studies are needed to highlight the relationship between sclerostin and mortality in kidney transplantation, as well as clinically relevant outcomes such as major adverse cardiovascular events. Lastly, whether metabolic actions of sclerostin (fat metabolism, insulin resistance etc.) impacts graft function and cardiovascular events needs further assessment.

The inhibitory role of sclerostin in bone formation is well known, but is also suggested that increased bone as reflected by increased osteocyte number also increase sclerostin synthesis. Furthermore, skeletal heterogeneity of sclerostin expression is probable, reflecting differences in mechanical loading between different skeletal sites [102]. Acute high doses of glucocorticoids or cumulative doses of glucocorticoids may also shape the relationship between sclerostin and bone in KTRs. As with other issues, the role of sclerostin on VC is not straightforward. Two hypotheses were postulated two explain the diverge findings regarding vascular calcification. One is suggesting sclerostin plays a direct (stimulating) role in VC and the other hypothesis suggest protective effect of sclerostin on vascular calcification were explained above (Fig. 4). Other potential explanations for these diverge findings include the heterogeneity of VC at different anatomical locations which may lead to different results regarding the association between sclerostin and the measured vascular outcomes. This vascular anatomy specific association is supported by studies reporting differences in the local expression of sclerostin depending on the anatomical location of blood vessels [103, 104]. Assuming that sclerostin has a similar role in the vascular wall as in bone (i.e., decreasing mineralization), blockage of sclerostin potentially stimulates mineralization, hence promoting VC in the vessel wall which should be treated as a warning signal [26]. Indeed, inhibition of sclerostin with romosozumab for treatment of osteoporosis showed increased cardiovascular risk although treated patients were older and take more cardiovascular medication (anticoagulants, antiplatelet agents, antihypertensives) and therefore were probably more likely to have cardiovascular events at baseline [105]. In stable coronary heart disease patients, high sclerostin increased all-cause mortality risk [106]. In patients receiving hemodialysis (HD) treatment, high sclerostin levels presented a trend toward higher risk for cardiovascular mortality [107]. On the other hand, in end-stage renal disease patients receiving treatment with HD or hemodiafiltration low sclerostin levels were associated with higher symptoms of coronary heart disease and rhythm disturbance [108]. Studies are also present showing that high sclerostin levels was found in patients with stroke compared to health controls [109, 110].

In KTRs, no data regarding safety of romosozumab is available. Thus the particular role of sclerostin specifically in VC and kidney transplantation requires further investigation [111]. Lastly, it needs to be determined whether there is interrelationship between acute rejection episodes and sclerostin levels [21].

## Conclusion

Sclerostin is an important molecule MBD. In KTRs, various studies have shown that sclerostin is associated with graft function, bone parameters, vascular calcification, and arterial stiffness although non-uniformly. However, we did not find any specific study investigating the relationship between sclerostin and hard cardiovascular outcomes such as myocardial infarction, heart failure, stroke or peripheral arterial disease in KTRs. Thus, although sclerostin has potential to be a marker of risk stratification in KTR, more studies including movement to surrogate to clinical outcomes are needed to determine if sclerostin has can be used as a clinically actionable marker of BM-CKD in KTRs. Specifically, large-scale prospective studies are needed to evaluate the impact of sclerostin on CKD–MBD and whether sclerostin can be used to monitor treatment response in BMD and cardiovascular disease in KTRs. This is important since performed studies are mostly observational and not interventional and the cause and effect relationship cannot be inferred. Moreover, it needs to be clarified whether inhibition of sclerostin as a clinical strategy in KTRs is feasible or the effects of such intervention increases cardiovascular risk.

## Data Availability

No new data were created or analyzed in this review.
